# PROTOCOL: Plastics in the food system: Human health, economic and environmental impacts. A scoping review

**DOI:** 10.1002/cl2.1033

**Published:** 2019-07-19

**Authors:** Joe Yates, Megan Deeney, Howard White, Edward Joy, Sofia Kalamatianou, Suneetha Kadiyala

**Affiliations:** ^1^ Faculty of Epidemiology and Population Health London School of Hygiene & Tropical Medicine London United Kingdom; ^2^ Campbell Collaboration New Delhi India

## 1 BACKGROUND

### 1.1 The problem, condition or issue

As of 2017, the running total of virgin plastics produced, since mass production of synthetic polymers began less than 70 years ago, was 8,300 million metric tons (Geyer, Jambeck, & Law, 2017); a fact that reflects the versatility of this remarkable group of materials which serve a vast range of important functions across various industries and sectors. However, there is growing evidence that the proliferation of plastic production and a reliance on these materials in the world economy, particularly in single‐use or disposable forms, is leading to detrimental environmental and ecosystem impacts at local and global scales, with potentially negative implications for human health (Barboza, Dick Vethaak, Lavorante, Lundebye, & Guilhermino, 2018; UNEP, 2018).

Packaging accounts for ~40% of all plastics produced since the 1950s, of which 41% is used specifically for food or beverages (Schweitzer et al., 2018). This statistic refers primarily to the latter stage of the food system in which food products are processed, marketed and transferred to consumers. However, plastics are also used extensively at other stages of the food system, for example in agricultural mulch, fishing nets and crates for transporting produce. Taken as a whole, the food system is likely to account for a much larger proportion of the world's reliance on plastics than its share in the use of plastic packaging alone.

Within the food system, plastics play an important beneficial role in food transportation, preservation, hygiene and safety, increasing the lifespan of foods, the length of value chains and contributing to food and nutrition security (Claudio, 2012). Therefore, it is important that these beneficial functions are not overlooked in the public and policy debates concerning this material, its uses and impacts. However, recent decades have seen a correlation between substantial increases in plastic food packaging and upward trends in food waste (Schweitzer et al., 2018), suggesting that while plastic packaging can preserve food, in itself this might not be sufficient to reduce wastage. Recent calls to action on plastics are driven in part by observations that the widespread utilisation of single‐use or disposable plastics, coupled with poor recycling rates and waste management is contributing to visible build ups of plastic across natural environments and oceans around the world. To illustrate this flow, of the total 6300 Mt of plastic waste produced by 2015, only 9% had been recycled or repurposed, with the remaining 91% either incinerated, placed into landfill or leaking into the natural environment (Geyer et al., 2017).

The extent of the impacts of plastic pollution is still largely unknown and remains to be adequately explored. Among the evidence now beginning to emerge is that which reveals an increasing presence of microplastics, nanoplastics and synthetic polymers in marine food chains, food products and the air we breathe (Karami et al., 2017; Lusher, McHugh, & Thompson, 2013; Smith, Love, Rochman, & Neff, 2018; Tyree & Morrison, 2018). Not surprisingly, negative consequences for human and planetary health are also now being hypothesised and investigated (Barboza et al., 2018; Smith et al., 2018). These interlinking concerns around sustainability come in addition to toxicology research pointing towards potentially harmful effects that chemicals or additives used in plastics may pose for humans (Gray, Rasanayagam, Engel, & Rizzo, 2017; Rancière et al., 2015) as well as the suggestion that plastic packaging could be encouraging unhealthy diets (Relton, Strong, & Holdsworth, 2012). On a broader scale, it can be said that plastics are also linked to global warming and climate change, as around 99% of plastic monomers are derived from fossil fuels, the supply and demand for which contributes to greenhouse gas emissions (GHGs). Yet in agricultural production, evidence also suggests that plastic sheeting can deliver environmental benefits such as reduced GHG emissions (Petersen et al., 2013). Taking such trade‐offs into account, there are growing calls for improved data and evidence to better understand and address the various effects of plastics, whilst developing alternatives — where necessary — for the functions they serve (Efferth & Paul, 2017; The Lancet Planetary Health, 2017).

A major challenge for research investigating the role and impacts of plastics is to establish causal links between specific sectoral or industry flows and the impacts — both beneficial and harmful — that they might be having on natural environments, human health and wellbeing. Without a better understanding of these linkages, underpinned by a comprehensive and robust scientific evidence base, attempts to preserve the benefits and mitigate the harmful effects of plastics will be hampered.

For this reason, we will conduct a systematic scoping review, looking at the impacts of plastics that are used specifically in or across the food system. This will be conducted in line with the Preferred Reporting Items for Systematic Reviews and Meta‐Analyses (PRISMA) Extension for Scoping Reviews, which advises that scoping reviews 'may examine the extent (that is, size), range (variety), and nature (characteristics) of the evidence on a topic or question' (Tricco et al., 2018). To do this, we will explore the food system's constitutive sub‐sectors — from 'farm to flush' — to examine the extent (volume of research), range (variety of exposure‐outcome relationships) and nature (study characteristics) of evidence for the impact of food system plastics on human health, food security and economics at the individual or household level and the environment.

### 1.2 The exposure

We characterise the exposure as plastic, including chemicals specifically emanating from the plastic exposure that are essential to its fundamental structure or functionality (i.e., phthalate plasticisers, or chemicals such Bisphenol A) used at any point, for any purpose, explicitly within the food system.

Categories of plastics are defined by the Society of the Plastics Industry (SPI) resin identification codes and will include: polyethylene terephthalate, high‐density polyethylene, polyvinyl chloride, low‐density polyethylene, polypropylene, polystyrene or styrofoam, miscellaneous plastics (includes: polycarbonate, polylactide, acrylic, acrylonitrile butadiene, styrene, fibreglass and nylon) (Sustainable Packaging Coalition, 2017).

In accordance with the Food and Agricultural Organisation of the United Nations (FAO) we define the food system as 'the entire range of activities involved in the production, processing, marketing, consumption and disposal of goods that originate from agriculture, forestry or fisheries, including the inputs needed and the outputs generated at each of these steps' (Food and Agriculture Organization of the United Nations, 2013).

Plastic in the food system is found in many different forms. For example, in agriculture plastic is used for mulching, growing tunnels, greenhouses and irrigation systems. In fishing, plastics are used in nets, lines and traps. Food and produce processing, storage and distribution use plastic in the form of packing crates, wrapping and food contact equipment. Packaging forms a crucial part of sales, marketing and consumption of goods. In addition, plastic food shopping bags, plastic crockery and cooking equipment are widely available at the consumer level. Finally, at the end of the food system with food disposal and waste management, plastic is used in pipes, compost storage containers, or in the black plastic bags thrown into landfill.

The durability of plastic means that its use within the food system may have both short and long term effects. For example, plastic mulch used in agriculture may increase crop yields in the short term, but when the plastic begins to break down in the soil, it may have an impact on plant growth or the soil microbiota for years to come (Steinmetz et al., 2016). Estimates of biodegradation times in marine environments for different plastics range from around 20 years for a plastic shopping bag, to 50 years for a styrofoam cup, to 450 years for a plastic bottle and up to 600 years for plastic fishing lines (Statistica & Grant, 2018). This means that the use of plastics within the food system may continue to have an effect on the environment, human health and well‐being, long after their intended function has passed. In this sense, the responsibility for these plastic products, and the impact that they have, must also endure.

### 1.3 How the intervention might work

Please note: For the purposes of this review, we use 'impacts' and 'outcomes' interchangeably.

This scoping review seeks to explore a range of exposure to outcome pathways, always beginning with plastics specifically used in the food system and ending at various outcome stages along three ultimate impact domains:

- human health.
- individual/household food security and economic factors.
- the natural environment.

**Figure 1:**
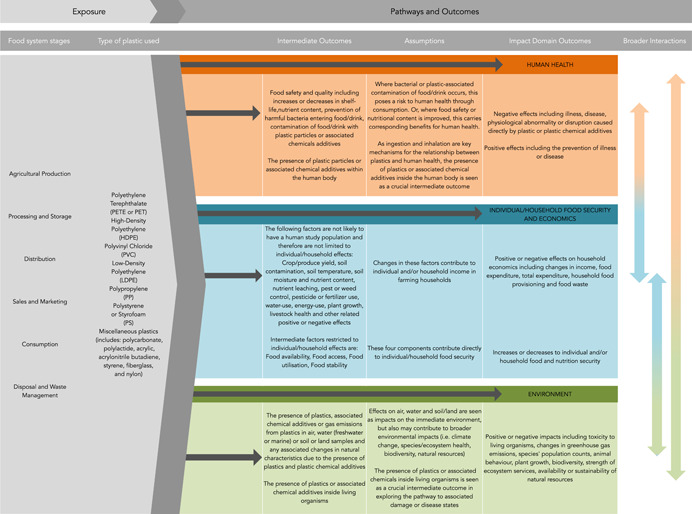
Logic model for this systematic scoping review on the impact of plastics used within the food system on human health, individual and household food security and economic factors and the natural environment [Color figure can be viewed at wileyonlinelibrary.com]

Given the interdisciplinary nature of this review, we expect heterogeneity among the specific outcomes considered in the literature, and therefore heterogeneity in the associated mechanisms and pathways to impact. For this reason, our logic model **(**Figure 1 **)** encapsulates the major anticipated relationships between exposures and outcomes whilst also providing the necessary flexibility for more defined pathways to be informed by the literature.

To maintain clarity, the three ultimate impact domains are considered as distinct outcome areas, though we acknowledge that the three domains are interlinked and often interdependent (Pan American Health Organization, 2013). We reflect these bi‐directional relationships in the 'Broader Interactions' of Figure 1.

The logic model depicts the relationship between the exposure (plastics in the food system) and the three ultimate impact domains. It encompasses potential direct outcomes (i.e., plastics in the food system directly leading to an ultimate impact domain), as well as intermediate outcomes that we reasonably hypothesise as being on the pathway to impact (i.e., plastics in the food system leading to impacts on a relevant population that exists prior to an ultimate impact domain). The grey arrows indicate these relationships that we seek to elucidate. Our assumptions regarding intermediate outcomes are stated in Figure 1.

#### 1.3.1 Impact domain 1: human health

We anticipate that existing literature could describe a relationship between any of the listed plastic types, used at any point of the food system, and a direct impact on human health. Studies considering this relationship will have to include human populations, demonstrating either a harmful effect or association (including contributing to illness, disease, physical or congenital abnormalities or physiological disruptions), a beneficial effect or association (including protection or promotion of health and the prevention of illness or disease), or null effect or association. The arrow from exposure direct to human health demonstrates this element of our enquiry.

Specific human health impacts that have been considered in the more general literature on plastics are intestinal damage and tissue abrasion from plastic particles themselves (Revel, Châtel, & Mouneyrac, 2018) and impacts via chemicals leached from the plastics on endocrine dysfunction, diabetes, and reproductive problems for example (Thompson, Moore, vom Saal, & Swan, 2009). In order to qualify for our scoping review, studies will have to demonstrate that the plastic, including chemicals leached from the plastic, originated from a specific human use within the food system, which is not always the case. Research has also considered the role of plastic food packaging in the protection of human health. For example, foodborne diseases (i.e., Salmonella) caused 420,000 deaths in 2010 (World Health Organization, 2015). Plastic packaging can help to protect human health by keeping food sterile and safe for consumption (Claudio, 2012), hence, studies showing a change in pathogen/bacterial content associated with food system plastics would be included in our review along the food safety pathway to human health.

Mechanisms, by which the relationship between plastics and human health exist include ingestion, dermal exposure or inhalation of plastic or plastic chemical additives (Revel et al., 2018; Thompson et al., 2009). In addition to the direct impacts on human health, we anticipate that certain studies will demonstrate effects or associations with intermediate outcomes, inferring a subsequent impact on human health as shown in Figure 1. An important intermediate outcome, as mentioned above, is food safety or contamination (World Health Organization, 2019). Some studies may demonstrate the presence (or absence) of known harmful bacteria and toxicants in foodstuffs or drink as a final outcome. If a food system plastic is implicated in this effect on the food or drink then we will reasonably assume the potential for onward effects on human health. Similarly, we will also consider studies that assess the presence (or absence) of plastic particles or associated chemical additives in food or drink items as an intermediate step on the pathway to human health outcomes. Between food safety and human health, we will consider one further intermediate step — the presence of plastic particles or associated chemical additives within the human body. Studies may not be able to provide evidence of an associated disease state given the potential difficulties in studying this pathway from food system plastics to proven disease, however, we wish to include crucial intermediate outcomes so as to fully capture the range of potential human health impacts. Regardless of the specific outcome, each study will need to demonstrate that the plastic exposure originated specifically within the food system.

#### 1.3.2 Impact domain 2: individual/household food security and economic factors

As a hugely complex and intricate field, our consideration of the impacts of plastics on food security and economics is focused on selected factors that do not attempt to describe all potential pathways or relationships between exposures and outcomes. Our key interest in this impact domain is individual and household level food and nutrition security, and selected economic factors including changes in income, food expenditure, total expenditure and household food provisioning and food waste. We anticipate that literature could describe a direct effect or association with the use of plastics in the food system on these outcome areas, which could be of a beneficial or harmful nature (Figure 1).

Due to the complexity of determinants of food and nutrition security and household economics, we are aiming to capture a range of intermediate outcomes that will be useful in examining this relationship. These include factors such as food availability, food access, food utilisation, food stability, crop or produce yield, livestock growth and welfare, soil contamination, soil temperature, soil moisture and nutrient content, nutrient leaching, pest or weed control, pesticide or fertiliser use, water‐use, energy‐use, plant growth, livestock health and other related beneficial, harmful or null effects or associations. We provide this range of outcome measures as a guide in advance of our screening process but a key focus of our scoping review is to gather the range of outcome indicators used in these fields and therefore we will remain flexible in updating our list of intermediate outcomes as we proceed. Our assumptions in linking these 'intermediate' outcomes to the impact domains are stated in Figure 1.

For example, plastic plays an important role in hygiene and preservation of food, in both transport and storage phases of the food system (Claudio, 2012). Logically, if plastic increases the hygiene and safety of food, along with its ability to travel undamaged, and its capacity to be stored for longer durations in the home or locally (Our Food: Public Health Impacts of Packaging), then plastic may increase food availability by increasing trade and transport possibilities, increasing food access in hard‐to‐reach regions, and increasing food stability and food utilisation — since people can store, process and preserve food safely, guarding themselves against shortages. These components are known as the 'Four Pillars of Food Security' (Bokeloh, Gerster‐Bentaya, & Weingärtner, 2005) and will be considered as 'intermediate' outcomes in accordance with general consensus on the building blocks of food security.

We also include factors such as crop/product yield and post‐harvest losses in the 'intermediate' outcomes under the assumption that changes at this level will have an effect on the income of agricultural families (Food and Agriculture Organization of the United Nations, 2014). Due to their multifactorial nature, we anticipate that studies may not follow through to looking at the impacts of plastic on household economy or income and therefore we include these intermediate outcomes in order to broaden our understanding of the potentially beneficial impacts of plastics.

#### 1.3.3 Impact domain 3: the natural environment

The health of the natural environment is multifaceted. It can be framed in terms of climate change, biodiversity, species health and population counts, ecosystem services and natural resources (OECD, 2003). We anticipate that literature could describe direct impacts of plastic used in the food system on any of these impact areas. This is shown in the Impact Domain column of Figure 1. The grey arrow indicates our line of exploration direct from exposure (plastics in the food system) to outcome (impact on the environment).

Due to the range of indicators that can be used to describe the natural environment, pathways from exposure to outcome will be similarly diverse. An example of the direct impacts of plastic is harm caused to marine species through ingestion and entanglement, leading to tissue abrasion, gut obstruction, dermal wounds and deaths (Duncan et al., 2017; Law, 2017). It has also been shown that the most common plastics used in the world are releasing greenhouse gases, methane and ethylene, into the air when exposed to ambient solar radiation. This includes plastics on the land and in the sea, no longer in use, but contributing to gas emissions and therefore to climate change (Royer, Ferrón, Wilson, & Karl, 2018). However, plastic sheets used to cover manure in the agricultural sector have also been shown to reduce the amount of methane released into the atmosphere, demonstrating an environmentally protective effect (Petersen et al., 2013). We aim to capture any measurable environmental changes that occur as a result of food system plastics.

As shown in Figure 1, we will also include impacts on the 'intermediate' outcomes such as the presence of plastic particles or chemical additives in soil, air and water (both freshwater and marine) samples. As essential components of the natural environment, any effects on soil, air or water constitute impacts on the environment in themselves. However, in our logic model, these are placed as 'intermediate' outcomes due to their importance in sustaining broader elements of the natural environment, including animal and plant life, the balance of ecosystems and climate. We anticipate that studies may consider effects at this intermediate level without being able to draw direct links to ecosystem damage or global warming for example, however we wish to include them in our review in order to capture the full range of environmental impacts. Similarly we wish to include as an intermediate outcome, the presence of plastics or associated chemical additives within living organisms as we view this as a crucial step on the pathway from plastic exposure to organism damage or disease states. These studies will have to demonstrate changes over time or before‐and‐after a change in exposure status in order to demonstrate changing impacts rather than prevalence studies.

### 1.4 Why is it important to do the review?

#### 1.4.1 Existing and ongoing primary research, narrative and systematic reviews, and meta‐analyses on the topic

Awareness and research of the impacts of our reliance on plastics has increased dramatically over the last 20 years and emanates from different fields of study such as agricultural production, food technology, materials science, toxicology, environmental health sciences and public health research. Due to rapid increases in research and the diversity of the fields, the evidence for the impact of food system plastic is piecemeal. An accurate understanding of the current state of research across these fields, focusing specifically on the impact of plastics used within the food system, will highlight areas for future research that can lead to evidenced‐based, targeted action and greater accountability among food system actors.

In just the last year, 2018, numerous primary research studies have been published, for example studies examining the impacts of plastic marine litter on coral, of plastic mulch on crop yield and land degradation, of mulch residue on future plant growth, and of plastic packaging on fruit juice contamination (Gao et al., 2019; Haque, Jahiruddin, & Clarke, 2018; Rastkari, Jeddi, Yunesian, & Ahmadkhaniha, 2018; Valderrama Ballesteros, Matthews, & Hoeksema, 2018). These are just a few of the more recent studies, and with many more appearing it is essential that we build a coherent and comprehensive picture of the research that has been done in order to highlight specific areas where there is a need for additional primary research, full systematic reviews or policy decisions.

Important literature reviews exist that look at the impacts of plastics in marine environments, on the soil ecosystem and on human health (Chae & An, 2018; Law, 2017; Revel et al., 2018). These reviews are crucial for elucidating knowledge on the impacts of plastics within specific outcome areas, however plastic is considered generally and emanates from different industry sources. In addition, these reviews are not conducted systematically. We aim to add to this understanding by linking impacts to a specific source of plastic usage (the food system and its sub‐sectors), in order to increase accountability. Additionally we aim to broaden the consideration of impact domains to simultaneously take into account the human health, food security and economic and environmental impacts of plastics.

Literature reviews, albeit not systematic, have addressed some impacts of specific uses of plastics in the food system, for example, a review of agricultural plastic mulching on soil quality (Steinmetz et al., 2016). These kinds of reviews are useful in offering depth to our understanding of the impacts of specific uses of plastics however they are narrow in scope, both at the exposure and outcome level, and can not address our aims of characterising the current research landscape on the impacts of plastics within the food system.

#### 1.4.2 Potential application of review findings

The effect that our reliance on plastic is having on our surroundings and on our own health has been a growing public concern in recent years. Governments around the world are implementing policies to reduce plastic usage across sectors, including the food sector, and to improve disposal and recycling methods (UNEP, 2018). Several policies in the form of nationwide bans and levies on the production, import and sales of polythene bags have been implemented globally. Amongst the first countries to formalise such policies were Denmark in 1994, Bangladesh in 2002, South Africa in 2003 and Tanzania in 2006 (UNEP, 2018). A ban on plastic bags was reinforced in Delhi, India in 2017, implemented with monetary fines for vendors and businesses caught using them, though the efficacy of this approach remains unclear (FE Online, 2017). Additional planned policies include a ban on plastic straws, cutlery and restrictions for non‐reusable coffee cups in the European Union (Batchelor, 2018). In order to ensure that policies are effective in reducing or reforming the use of plastics, and are sufficiently robust in meeting resistance from the plastics industry, a strong evidence‐base is required (UNEP, 2018). Our scoping review will contribute to providing an evidence‐base for sustainable food system policies by delivering a broad picture of existing evidence pertaining to the impacts — beneficial, harmful and null — of plastics used in the food system on human health, individual and household food security and economics and the environment.

## 2 OBJECTIVES

This scoping review is motivated by the following questions:

What is the extent, range and nature of the evidence on the human health, individual and household food security and economics, and environmental impacts of plastics used within the food system, including the extent to which research has been conducted, the range of exposure‐outcome relationships considered and study design characteristics in this field, since 2000?

Are there evidence gaps or shortfalls relating to the impacts of plastics in the food system and where is there a need for further primary research or opportunities to conduct systematic reviews within the context of the human health, individual and household food security and economics, and environmental impacts of plastics in the food system?

## 3 METHODOLOGY

The methodology for our scoping review is informed by the PRISMA Extension for Scoping Reviews checklist (Tricco et al., 2018) and will examine the extent, range and nature of the evidence for the impact of plastics used within the food system on human health, food security and economics at the individual or household level, and the environment.

Screening will be conducted in two stages, first by title and abstract and secondly by full text. Four researchers will be double screening at title and abstract stage. We held an initial training session via an online meeting with screening tools provided to each screener along with 10 example articles. Screeners were blind as to the inclusion/exclusion status of these articles and training was completed through discussion and on‐the‐spot tests. We then completed 3 rounds of 100 randomly selected articles that were double screened by Joe Yates or Megan Deeney. Discrepancies were resolved by Megan Deeney and additional in‐depth training sessions and/or notes on discrepancies were provided to screeners after each round, before moving on to the next.

Discrepancies in inclusion/exclusion decisions during title and abstract phase will be resolved by automatically including those results for full text review. Additional training will be provided ahead of full text screening and agreement rates will be calculated through double screening subsets of 20–30 results at a time, until a consistent average agreement rate of above 80% is achieved. Full texts will then be single screened with 5% checks by Joe Yates. Coding for data extraction purposes will be trained for and tested in the same way.

### 3.1 Criteria for including and excluding studies

#### 3.1.1 Types of study designs

We will include all experimental studies, and non‐experimental studies including: analytical cross sectional (single time point with a comparator group), repeated cross sectional (multiple time points with or without comparator group), longitudinal observational cohort case‐control, case study (post‐mortem, diagnosis of illness, injury or entanglement) ecological (including temporal and geographical), modelling (including risk assessments and life cycle assessments) and case studies in which the outcome is a clear diagnosis of cause‐of‐death, illness, injury or entanglement.

Descriptive cross‐sectional studies in which there is no comparator group or time‐point comparison will be excluded as will case studies that do not report a clear diagnosis of cause‐of‐death, illness, injury or entanglement. Qualitative studies will be excluded.

#### 3.1.2 Types of participants (detailed by outcome category)

##### Impact domain 1: human health

###### Impact outcomes (harmful effects or associations including illness, disease, physiological abnormality or disruption caused directly by plastic or plastic chemical additives; beneficial effects or associations including the prevention of illness or disease and null effects or associations)

Studies of the impact of plastics in the food system on human health may refer to human populations in any geographical location, including high, middle and low‐income countries, in urban and/or rural areas. Studies conducted between 2000‐present will be included, those before 2000 will be excluded. This is to take into account the dynamic nature and evolution of food systems, coupled with considerable increases in plastic production and associated waste since 2000 (Geyer et al., 2017). More detail on date restrictions can be found further in this protocol. Animal experiments will be excluded.

###### Intermediate human health outcomes

Studies looking at the impact of plastics used in the food system on food safety, shelf life, nutrient content and contamination or related outcomes will not necessarily include a human population. Instead the study population will consist of food or drink samples.

Studies investigating plastics used within the food system and their subsequent presence (or lack of) in the human body may refer to human populations in any geographical location, including high, middle and low‐income countries, in urban and/or rural areas. The same date restrictions will be applied to intermediate outcome populations (publication date: 2000 onwards).

##### Impact domain 2: individual/household food security and economics

###### Impact outcomes (increases or decreases to individual and/or household food and nutrition security and beneficial, harmful or null effects or associations with household economics including changes in income, food expenditure, total expenditure, household food provisioning and food waste)

Studies of the impact of plastics in the food system on individual or household food security and economics may refer to human populations at the individual or household level in any geographical location, including high‐ middle and low‐income countries, in urban and/or rural areas, from 2000 to present. Studies that consider food security or economics on a broader scale such as at industry or national levels will be excluded.

###### Intermediate food security/economic outcomes

1. Food availability, food access, food utilisation and food stability: Studies of the impact of plastics used in the food system on the four pillars of food security may refer to human populations at the individual or household level in any geographical location, including high‐ middle and low‐income countries, in urban and/or rural areas, from 2000 to present. Studies that consider these outcomes at a broader scale such as at industry or national levels will be excluded.
2. Crop/produce yield, soil contamination, soil temperature, soil moisture and nutrient content, nutrient leaching, pest or weed control, pesticide or fertiliser use, water‐use, energy‐use, plant growth, livestock health and other related beneficial, harmful or null effects or associations. It is possible that studies of the impact of plastics used in the food system on these outcomes may not refer to a human population. Instead, we anticipate that the study population will be the crop, livestock, produce or soil sample to which these outcomes relate.

##### Impact domain 3: the natural environment

###### Impact outcomes: (Beneficial, harmful or null effects or associations including toxicity to living organisms, changes in greenhouse gas emissions, species’ population counts, animal behaviour, plant growth, biodiversity, strength of ecosystem services, availability or sustainability of natural resources

Studies of the impact of plastics in the food system on the environment may refer to animal, plant or bacterial populations or natural resources in terrestrial, aquatic and/or aerial environments, from 2000 to present.

###### Intermediate environmental outcomes

1. Presence of plastics, associated chemical additives or gas emissions in water, soil or the air: Studies will be included if the study population comprises soil, water or air samples, and as such refers to the natural environment in which humans, animals, plants and bacteria survive.
2. Presence of plastics or associated chemical additives inside living organisms: Study populations may include animal, plant or bacterial populations in terrestrial, aquatic and/or aerial environments, from 2000 to present.

#### 3.1.3 Types of exposures

The exposure is plastic used at any point within the food system, as well as chemical additives that are essential for its fundamental structure or functionality (i.e., phthalate plasticisers, or chemicals such Bisphenol A). In accordance with the FAO we define the food system as 'the entire range of activities involved in the production, processing, marketing, consumption and disposal of goods that originate from agriculture, forestry or fisheries, including the inputs needed and the outputs generated at each of these steps' (Food and Agricultural Organization of the United Nations, 2013). We will include studies related to the system surrounding any human dietary component. For example: meat, poultry and fish, fruit and vegetables, grains, legumes and pulses, fats and oils, processed foods and confectionery, herbs and spices, edible flowers. We will also include studies that use food and drink simulants to show an effect or null effect of food system plastics or their chemical additives. Alcohol, packaged or bottled water and soft drinks will also be included. We will exclude studies related to the production and other human activities around tobacco, ornamental plants, forestry for timber and other non‐dietary produce. We will exclude studies in which the source of plastic is industries outside of the food system, for example in medicine or cosmetics.

Specific key terminology for different forms of plastic is taken from SPI resin identification codes and will include: polyethylene terephthalate, high‐density polyethylene, polyvinyl chloride, low‐density polyethylene, polypropylene, polystyrene, acrylic, polycarbonate, polylactic fibres, nylon and fibreglass (Sustainable Packaging Coalition, 2017). These plastic categories will direct the search strategy terms. Literature that refers only to macro, micro or nanoplastics that cannot be identified as specifically originating from use within the food system will be excluded.

#### 3.1.4 Comparison groups

Comparison groups will include study population groups exposed to:

- No plastic (control)
- Less plastic (e.g., quantity/thickness of the same material)
- Different material (e.g., paper, jute, straw)
- Different type of plastic
- Pre‐exposure to plastic (compared with post exposure) in time
- Multiple time points of varying levels of plastic exposure
- No comparator group or comparison in time (only permissible with case studies of entanglement/injury/illness/death)

#### 3.1.5 Types of outcome measures

Our scoping review is intended to identify and summarise the state and current trends of research on the impact of plastics used within the food system on three selected overarching outcome domains: human health, food security and economic impacts at the individual or household level and environmental impacts at any level. Our review aims to capture and characterise the breadth of specific impact indicators that have been explored in recent literature, for this reason we are not a priori restricting the outcome indicators used within these three domains.

Measures will be included in the review provided that they fall within our broad definitions of outcome categories. The following list provides examples of expected outcomes but this will be updated as we uncover outcomes from the literature during screening

1. Human health: Harmful associations or effects including illness, disease, physiological abnormality or disruption caused directly by plastic or plastic chemical additives, beneficial associations or effects including the prevention of illness or disease or null effects or associations. Additionally, measures may fall within the definitions of our selected intermediate outcomes including food safety and quality including increases or decreases in shelf‐life, nutrient content, prevention of harmful bacteria entering food/drink, contamination of food/drink with plastic particles or associated chemicals additives and the presence of plastics or associated chemical additives within the human body
2. Individual/household food security and economics: Increases or decreases to individual and/or household food and nutrition security and beneficial, harmful or null effects or associations with household economics including changes in income, food expenditure, total expenditure, household food provisioning and food waste. Additionally, measures may fall within the definitions of our selected intermediate outcomes including food availability, food access, food utilisation, food stability, crop/produce yield, soil contamination, soil temperature, soil moisture and nutrient content, nutrient leaching, pest or weed control, pesticide or fertiliser use, water‐use, energy‐use, plant growth, livestock health and other related beneficial, harmful or null effects or associations.
3. The natural environment: Beneficial, harmful or null effects or associations including toxicity to living organisms, changes in greenhouse gas emissions, species’ population counts, animal behaviour, plant growth, biodiversity, strength of ecosystem services, availability or sustainability of natural resources. Additionally, measures may fall within the definitions of our selected intermediate outcomes including the presence of plastics or their associated chemical additives in soil, air or water samples and inside living organisms.

#### 3.1.6 Duration of follow‐up

There will be no restrictions placed on the duration of follow‐up of primary research. Eligible study designs include cross‐sectional, modelling and case study designs, which do not involve follow‐up.

#### 3.1.7 Types of settings

There will be no restrictions placed on the setting of primary research. Settings may therefore include terrestrial, aquatic or aerial environments, in any part of the world.

#### 3.1.8 Date restrictions

In order for this review to be useful for researchers and policymakers we will capture literature from the year 2000 onwards. This is for a number of key reasons:

1. Food system evolution in the context of globalisation
  Since 2000, the forces of globalisation, consumerism and population growth have driven fundamental evolutions in our food system. These step changes, supported increasingly by the expansion of internet technologies, mean that modern value chains are longer than ever, thus requiring innovations around food production, processing, transportation, preservation, safety and hygiene. Plastic has played a critical role in this recent evolution (i.e., in preserving food safely and maintaining its quality during transportation). It has also in part facilitated a shift towards diets constituted from highly processed foods. We expect to find the most relevant literature for each of these in the years from 2000 onwards during which these changes occurred.
2. Regulatory and policy environment
  As mentioned in the section on 'Potential application of review findings', waste management, recycling systems and policies around disposable plastic are still in their relative infancy and have evolved significantly during the past 20 years. Despite the large growth in plastic usage and cumulative evidence of associated environmental problems, the first national plastic bag ban was only introduced in 2002 (by Bangladesh). In 2000–2001 key steps were taken to make recycling more simple at the consumer level in countries like the USA, which resulted in substantial increases in recycling rates. The regulatory environment around food safety has also changed since the late 1990s. For example, the Food Quality Protection Act — which primarily addressed pesticide use, but also encapsulated more modern thinking around toxicity and antimicrobial reform — was only introduced in USA in 1996. Similarly, the European Food Safety Authority was established in 2002.
3. Plastics supply and demand
  Reflecting the aforementioned forces of globalisation, consumption and population growth, the production and use of plastics has increased radically since 2000. In 1976, 50 m tonnes of plastic were produced annually, whereas in 2002 this figure was 200 m and in 2015; 322 m (Statista, 2018). The scale of plastic use and its potential for exerting beneficial and harmful impacts in today's context is therefore altogether different from pre‐2000.
4. Technological change and scientific understanding
  The last two decades have seen rapid technological advances underpinning not only the food system and recycling capabilities, but also new biodegradable materials and alternatives to fossil‐fuel derived plastic polymers. Alongside this, there has been an increased understanding of impact domains, supported by new metrics and tools to measure these phenomena. For example, there is now a far more nuanced enhanced understanding of dose‐response relationships than existed in the 1990s and more sophisticated tools by which to measure toxicity in food and humans e.g. in the case of BPA (Vogel, 2009 *)*. Together, this means that data from this period are most relevant for our review.

We will offer a brief reflection of this limitation in the discussion section of the paper.

#### 3.1.9 Search strategy

The search strategy has been devised with the involvement of a search specialist and extensive trial searches, tested against key texts. The following search string will be applied in the stated literature databases and the same key search terms will be used to search for grey literature.

Example search string applied to Scopus:

((TITLE‐ABS‐KEY (agri* OR agro* OR farm OR farms OR farming OR aquacultur* OR aquafarm OR aquafarming OR aquatic OR grain OR grains OR cereal OR cereals OR legume OR legumes OR leguminous OR pea OR peas OR bean OR beans OR lentil OR lentils OR fish* OR poultry OR egg OR eggs OR confectionery OR vegetable* OR fruit OR fruits OR livestock OR meat OR dairy OR seafood OR food* OR drink* OR beverage* OR "potable water" OR "bottled water" OR coffee OR tea OR grocery OR groceries OR snack OR snacks OR meal OR meals OR supermarket* OR "local market" OR "local markets" OR "fast‐food" OR "fast food" OR "take‐away" OR takeaway OR catering OR restaurant* OR "fats and oils" OR "cooking oil" OR "sunflower oil" OR "olive oil" OR "palm oil" OR "coconut oil" OR nut OR nuts)) AND (TITLE‐ABS‐KEY (plastic OR plastics OR plasticulture OR macroplastic* OR mesoplastic* OR microplastic* OR nanoplastic* OR microfiber* OR microfibre* OR polyethylene OR "polyvinyl chloride" OR polypropylene OR polystyrene OR acrylic OR polycarbonate OR polylactide OR "polylactic acid" OR styrofoam OR styrene OR "acrylonitrile butadiene" OR nylon OR fibreglass OR fiberglass OR phthalate* OR bisphenol*) AND NOT (fiber‐optic OR fibre‐optic OR fiberoptic OR fibreoptic OR prosthetic* OR prosthesis OR "plastic surgery" OR "plastic scintillation" OR "plastic scintillator" OR "plastic scintillating" OR metallurg* OR "grain boundaries")) AND (TITLE‐ABS‐KEY ("human health" OR nutri* OR diet* OR "food safety" OR foodborne OR "food borne" OR "food‐borne" OR illness* OR disease* OR disorder* OR abnormal* OR gene OR genes OR genetic OR dna OR digestion OR digestive OR gastrointestinal OR "gastro‐intestinal" OR "nervous system" OR reproduction OR reproductive OR (circulation W/2 blood) OR "circulatory system" OR neural OR endocrine OR lymphatic OR "respiratory system" OR respiration OR fertility OR "birth defect" OR toxic* OR environment* OR contamina* OR ecology OR ecosystem* OR habitat OR habitats OR biodiversity OR flora OR fauna OR animal OR animals OR bird OR birds OR insect OR insects OR coral OR "plant health" OR "plant growth" OR "tree health" OR bacteri* OR microb* OR soil OR "air quality" OR "water quality" OR marine OR ocean* OR sea OR seas OR lake OR lakes OR river OR rivers OR waterway OR waterways OR "surface water" OR pollut* OR "land degradation" OR "greenhouse gas*" OR "gas emissions" OR "climate change" OR "climate‐change" OR "global warming" OR "climate warming" OR "greenhouse effect" OR "climate variability" OR "resource depletion" OR "depletion of natural resources" OR dioxins OR "carbon‐dioxide" OR "carbon dioxide" OR "carbon monoxide" OR "carbon‐monoxide" OR "chemical leaching" OR "energy saving*" OR "energy‐saving*" OR "water saving*" OR "water‐saving*" OR income OR wage OR wages OR "food waste" OR "crop yield" OR "fish yield" OR "milk yield" OR "meat yield" OR "livestock yield" OR "poultry yield" OR "crop loss" OR "post‐harvest loss" OR "postharvest loss" OR "food expenditure" OR "total expenditure" OR "household food provisioning" OR "household expenditure" OR "household economy" OR "cost saving*" OR "cost‐saving*" OR "household economics" OR "nutrition security" OR (food W/2 security) OR (food W/2 insecurity) OR (food W/2 preservation) OR (food W/2 access) OR (food W/2 availability) OR (food W/2 utilisation) OR (food W/2 utilization) OR (food W/2 stability) OR (food W/2 surplus) OR (food W/2 shortfall) OR (food W/2 spoilage) OR (water W/2 access) OR (water W/2 safety) OR ("land use" W/2 change) OR (land‐use W/2 change)))) AND (LIMIT‐TO (PUBYEAR, 2019) OR LIMIT‐TO (PUBYEAR, 2018) OR LIMIT‐TO (PUBYEAR, 2017) OR LIMIT‐TO (PUBYEAR, 2016) OR LIMIT‐TO (PUBYEAR, 2015) OR LIMIT‐TO (PUBYEAR, 2014) OR LIMIT‐TO (PUBYEAR, 2013) OR LIMIT‐TO (PUBYEAR, 2012) OR LIMIT‐TO (PUBYEAR, 2011) OR LIMIT‐TO (PUBYEAR, 2010) OR LIMIT‐TO (PUBYEAR, 2009) OR LIMIT‐TO (PUBYEAR, 2008) OR LIMIT‐TO (PUBYEAR, 2007) OR LIMIT‐TO (PUBYEAR, 2006) OR LIMIT‐TO (PUBYEAR, 2005) OR LIMIT‐TO (PUBYEAR, 2004) OR LIMIT‐TO (PUBYEAR, 2003) OR LIMIT‐TO (PUBYEAR, 2002) OR LIMIT‐TO (PUBYEAR, 2001) OR LIMIT‐TO (PUBYEAR, 2000)) AND (LIMIT‐TO (LANGUAGE, "English"))

We will apply the systematic search strategy to the following databases:

- Agris
- CAB Abstracts
- CAB Global
- Campbell Library
- Cochrane Database of Systematic Reviews
- Epistemonikos
- GreenFile
- Web of Science
- Scopus

All searches will be conducted in English. Search limits will be set for language (English language only) and year of publication (≥ 2000 only).

Grey literature will be sought via the following sources:

- CGIAR research programme libraries
- European Commission
- International Food Policy Research Institute (IFPRI)
- World Health Organisation
- Food and Agriculture Organisation of the United Nations
- The World Bank
- The World Resource Institute
- Green Climate Fund
- Global Environment Facility
- United Nations Evaluation Group
- Department for Environment, Food and Rural Affairs (UK)
- United States Environmental Protection Agency (US)
- Stockholm Environment Institute
- European Environment Agency
- Institute for European Environment Policy

The same date and language restrictions will be applied to grey literature searches.

Literature eligible for inclusion will be hand‐searched for additional references. Relevant experts in the field of food systems, plastics and the natural environment will be contacted to highlight any key papers. In particular, we will liaise with a range of material and plastics experts at different points throughout the review where necessary to clarify technical queries outside the knowledge set of our team and to retrieve any relevant research these groups may have access to.

#### 3.1.10 Description of methods used in primary research

As a scoping review, this study aims to capture the breadth of methods that are being employed to study the impact of plastic used in the food system on the three specified outcome domains. From initial scoping searches we anticipate a broad range of methods, including experimental and observational studies, using varied forms of statistical analysis.

#### 3.1.11 Criteria for determination of independent findings

As a scoping review, albeit systematic, we aim to characterise the research landscape rather than appraise the quality of specific evidence or interventions.

#### 3.1.12 Details of study coding categories

**Figure 2:**
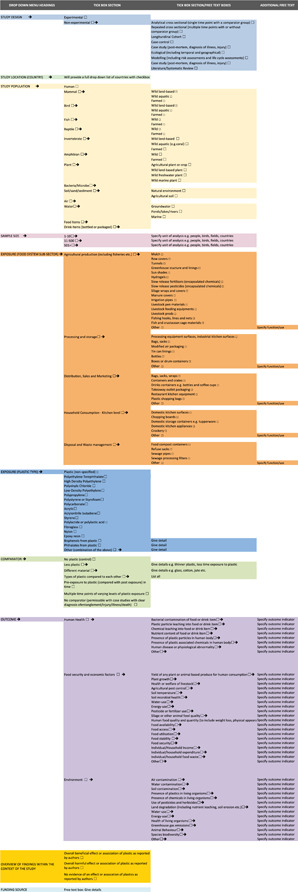
Coding tool for data extraction [Color figure can be viewed at wileyonlinelibrary.com]

Coding for data extraction will be completed on Eppi Reviewer software, using a mixture of checkboxes (indicated below) leading to further checkboxes or free text boxes. This will allow for consistency across coders with the necessary degree of flexibility to ensure that we are able to capture the broadest range of impacts as they emerge from the literature. A full coding structure is provided which will be updated at the outcome level as we progress through the screening (Figure 2).

#### 3.1.13 Statistical procedures and conventions

As a scoping review, summary statistics will be provided for the quantity of evidence by exposure and outcome category and any other emerging themes. Further statistical analyses will not be conducted for this review.

#### 3.1.14 Treatment of qualitative research

We do not plan to include qualitative research.

## CONFLICT OF INTERESTS

Howard White is CEO of the Campbell Collaboration for which there is an interest in increasing publications in the Campbell Library. However, he is not involved in the editorial process or decisions regarding the publication of this review. Authors have no other conflict of interests.

## AUTHOR CONTRIBUTIONS

Content: J.Y., M.D., S.K., E.J., S.K., Systematic review methods: S.K., H.W. Statistical analysis: N/A. Information retrieval: J.Y., M.D., S. K.
